# Chemical shift assignments of calmodulin under standard conditions at neutral pH

**DOI:** 10.1007/s12104-022-10082-7

**Published:** 2022-04-23

**Authors:** Aritra Bej, James B. Ames

**Affiliations:** 1 Department of Chemistry, University of California, Davis, CA 95616, USA

**Keywords:** CaM, Calcium, EF-hand, Chemical shift perturbation, NMR

## Abstract

The Ca^2+^ sensor protein, calmodulin (CaM) is ubiquitously expressed in all cells where it binds to hundreds of different target proteins, including dozens of enzymes, receptors, ion channels and numerous Ca^2+^ transporters. The only published NMR chemical shift assignments for Ca^2+^-bound CaM (in the absence of a target) have been determined under acidic conditions: at pH 6.5/310 K (BMRB 6541) and pH 6.3/320 K (BMRB 547). However, some CaM/target complexes are not soluble under these conditions. Also, amide chemical shifts are very sensitive to pH and temperature, which can cause large baseline errors when using the existing chemical shift assignments of free CaM to calculate chemical shift perturbations caused by target binding at neutral pH and physiological temperature. We report complete NMR chemical shift assignments of Ca^2+^-saturated CaM under a set of standard conditions at neutral pH and 308 K that will enable more accurate chemical shift comparison between free CaM and CaM/target complexes (BMRB 51289).

## Biological context

CaM is a soluble Ca^2+^ sensor protein (16.7 kDa) that belongs to the EF-hand superfamily of Ca^2+^ sensors (Ikura 1996, Moncrief et al. 1990). CaM contains four EF-hand motifs (EF1, EF2, EF3 and EF4) grouped into two independent domains (called the N-lobe and C-lobe). In the CaM crystal structure, EF1 and EF2 interact to form the CaM N-lobe while EF3 and EF4 interact to form the CaM C-lobe (Babu et al. 1988). The CaM N-lobe and C-lobe each bind two Ca^2+^ with positive cooperativity (Gilli et al. 1998). The Ca^2+^-saturated form of CaM is known to bind to hundreds of different target proteins, including dozens of enzymes, receptors, ion channels and other Ca^2+^ transporters (Ikura 1996). The Ca^2+^-induced binding of CaM to its various target proteins usually serves to augment the biological activity of the target protein. In some cases, CaM can act as both an activator and inactivator of the same target protein (Ames 2021). For example, the Ca^2+^-free form of CaM is believed to interact with and activate the L-type voltage gated Ca^2+^ channel (CaV1.2) under basal conditions (Adams et al. 2014; Ben Johny et al. 2013; Findeisen et al. 2013), whereas Ca^2+^-bound CaM interacts with and inactivates L-type Ca^2+^ channels in a process known as Ca^2+^-dependent inactivation (CDI) (Peterson et al. 1999; Zuhlke et al. 1999).

CaM is believed to bind to more than 200 different target proteins, and atomic-level structures are known for at least 30% of the known target complexes (Hoeflich and Ikura 2002). The vast majority of these structures involve Ca^2+^-bound forms of the CaM N-lobe and C-lobe that each bind to opposite sides of a target helix in a collapsed structure as seen in the structures of CaM bound to the myosin light chain kinase (MLCK) (PDB: 1CDL), CaM kinase II (PDB: 1CDM), CaM kinase kinase (PDB: 1CKK), ryanodine receptor 1 (PDB: 2BCX), ryanodine receptor 2 (PDB: 7KL5), CaV1.2 IQ-motif (2F3Y) and many other target proteins. By contrast, the two lobes of CaM are also known to each bind to separate target binding sites in a bipartite fashion as seen for CaM bound to the estrogen receptor-α (Zhang et al. 2012) and the Na^+^/H^+^ exchanger (NHE1) (Sjogaard-Frich et al. 2021). Lastly, CaM binding to the anthrax adenylate cyclase exotoxin can lead to a complete remodeling of the target complex (Drum et al. 2002).

NMR spectroscopy is a powerful method for analyzing and discovering the structures of CaM/target complexes. Indeed, the very first structure (CaM/MLCK) was solved by NMR (Ikura et al. 1992). NMR chemical shift perturbation analysis (difference in chemical shift caused by target binding) is frequently used to identify residues in CaM that interact with target proteins (called chemical shift mapping). Accurate chemical shift perturbation analysis requires the NMR spectrum of free CaM to be obtained under the same solvent conditions as the spectrum of the CaM/target complex. However, the only published chemical shift assignments of Ca^2+^-bound CaM (in the absence of a target) were obtained under acidic conditions (Ikura et al. 1990; Kainosho et al. 2006), but some CaM/target complexes are not soluble or stable under these conditions. Also, amide chemical shifts are very sensitive to pH, which can cause large baseline errors when comparing chemical shift values of free CaM at acidic pH with chemical shifts of CaM bound to a target at neutral pH. We report here NMR resonance assignments of Ca^2+^-saturated CaM under a set of standard conditions at neutral pH. These assignments along with future NMR studies of CaM/target complexes performed under standard conditions should enable a more systematic analysis of chemical shift perturbation and more accurately guide the discovery of new target interactions.

## Methods and experiments

### Expression and purification of CaM

Recombinant human CaM was subcloned into pET11b expression vector (Novagen) and overexpressed in *E. coli* strain BL21(DE3) as described previously (Turner et al. 2020). Uniformly ^13^C/^15^N-labeled CaM samples were overexpressed in M9 minimal media, containing 1 g/L ^15^NH_4_Cl and 3 g/L ^13^C-labeled glucose (Cambridge Isotopes Laboratories) as the sole nitrogen and carbon sources, respectively. The soluble fraction of the cell lysate was loaded onto a HiPrep Phenyl Sepharose 6 column that was pre-equilibrated with equilibration buffer, containing 20 mM Tris (pH 7.5), 200 mM KCl, 2 mM CaCl_2_. The CaM protein was eluted from the column using a buffer that contained 20 mM Tris (pH 7.5), 50 mM KCl, 2 mM EGTA. The eluted protein fraction was further loaded onto a HiPrep Q Sepharose anion exchange column that was pre-equilibrated with 50 mM Tris (pH 7.5), 25 mM KCl, 1 mM EGTA and eluted by a KCl gradient up to 625 mM. The purity and identity of the eluted protein fractions were confirmed by sodium dodecyl sulfate–polyacrylamide gel electrophoresis.

### NMR spectroscopy

**Fig. 1:**
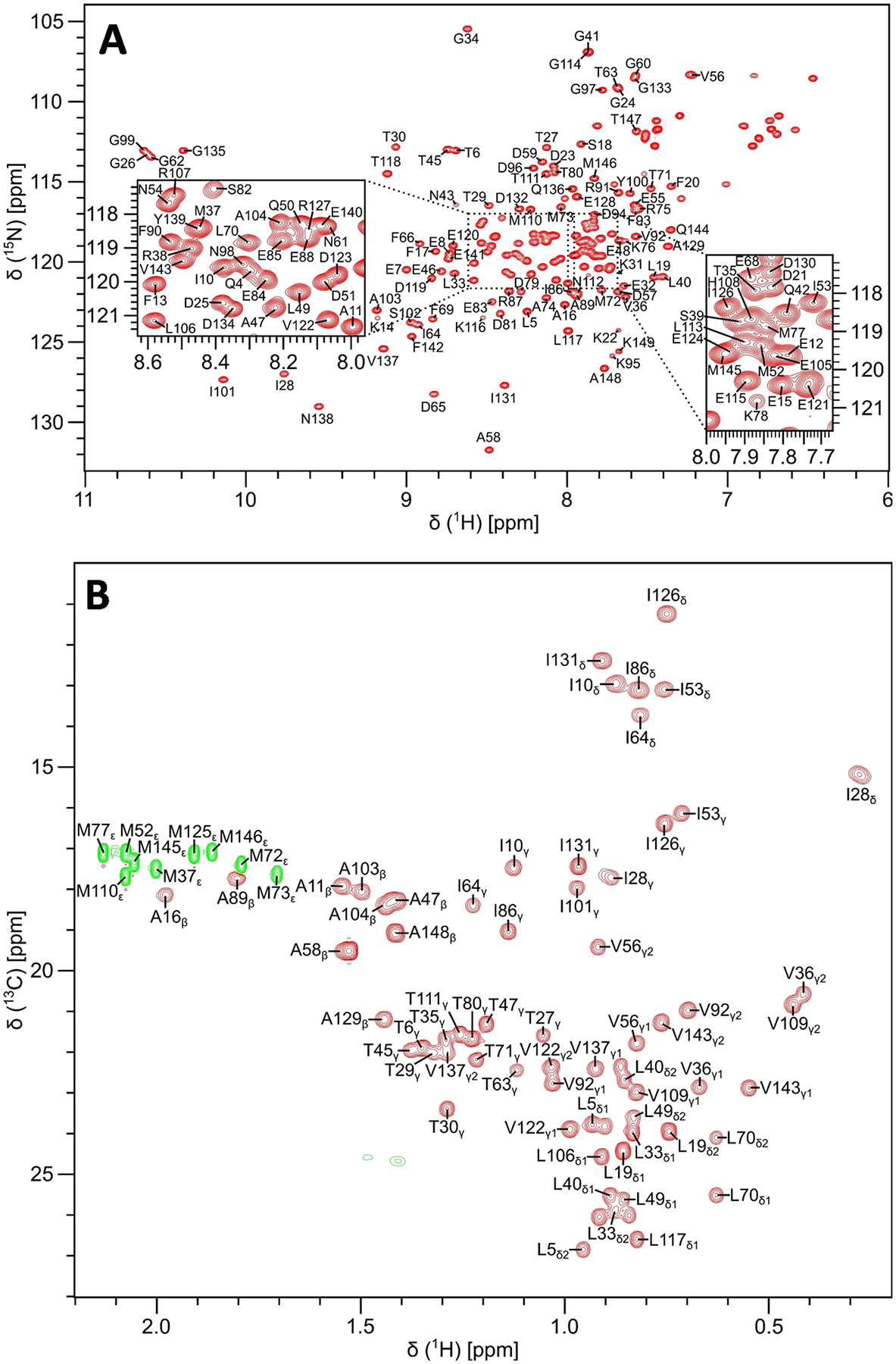
Two-dimensional NMR spectra of CaM under standard conditions at neutral pH. **A** ^15^N–^1^H HSQC spectrum recorded at 600 MHz ^1^H frequency was analyzed to determine backbone resonance assignments. The spectrally crowded regions are highlighted with black-dashed boxes (see inset). **B** Constant-time ^13^C–^1^H HSQC spectrum was analyzed to determine aliphatic side chain resonance assignments. Representative resonance assignments are indicated by residue labels; complete assignments are available as BMRB accession no. 51289

Protein samples of ^13^C/^15^N-labeled CaM were exchanged into a standard NMR buffer containing 20 mM Tris-d_11_ (pH 7.0) with 1 mM CaCl_2_, and 92% H_2_O/8% D_2_O. Many CaM/target complexes (MLCK, CaMKK, CaMKII, and IQ-motif) have optimal solubility under these standard conditions. The low ionic strength did not cause protein aggregation and was necessary to have optimal NMR sensitivity and cryoprobe performance. The Ca^2+^-bound CaM protein was concentrated to give a final concentration of 0.5 mM in a final volume of 0.3 mL. All NMR experiments were performed at 308 K on a Bruker Avance III 600 MHz spectrometer equipped with a four-channel interface and triple resonance cryogenic (TCI) probe. The ^15^N–^1^H HSQC spectrum (Fig. 1A) was recorded with 256 × 2048 complex points for ^15^ N(F1) and ^1^H(F2). Assignment of backbone resonances was obtained by analyzing the following spectra: HNCACB, CBCA(CO)NH, HNCO and HBHA(CO)NH. The assignment of side chain (aliphatic (Fig. 1B) and aromatic) resonances was obtained by analyzing the following spectra: HCCCONH-TOCSY, HCCH-TOCSY, HBCBCGCDHD and HBCBCGCDCEHE as described previously (Ikura et al. 1991). The NMR data were processed using NMRPipe and analyzed using Sparky.

## Extent of assignments and data deposition

Figure 1A presents the ^15^N–^1^H HSQC spectrum of Ca^2+^-bound CaM (hereafter CaM) under standard conditions at neutral pH to illustrate representative backbone resonance assignments. Figure 1B presents a constant-time ^13^C–^1^H HSQC spectrum to illustrate side chain methyl resonance assignments. The NMR assignments were based on 3D heteronuclear NMR experiments performed on ^13^C/^15^N-labeled CaM at neutral pH. The NMR spectra of CaM exhibited well-dispersed peaks indicative of a stably folded structure. Four amide resonances (assigned to G26, G62, G99 and G135) exhibited noteworthy downfield shifts that are caused by Ca^2+^ binding to each of the four EF-hands (Fig. 1A). Ring current shifted methyl resonances assigned to residues I28, V36, and V109 (Fig. 1B) are consistent with these methyl groups interacting with aromatic residues in the hydrophobic core as seen in the crystal structure. The percentage of assigned backbone resonances are as follows: ^1^HN (97%: 144 out of 149), ^15^ N (97%: 144 out of 149), ^13^Cα (98%: 146 out of 149), ^13^Cβ (91%: 135 out of 149), and ^13^CO (95%; 141 out of 149). The unassigned non-proline residues were M1, A2, and D3. More than 85% of aliphatic and aromatic side-chain resonances were assigned. The chemical shift assignments (^1^H, ^15^N, ^13^C) for CaM at neutral pH have been deposited in the BioMagResBank (http://www.bmrb.wisc.edu) under accession number 51289.

**Fig. 2:**
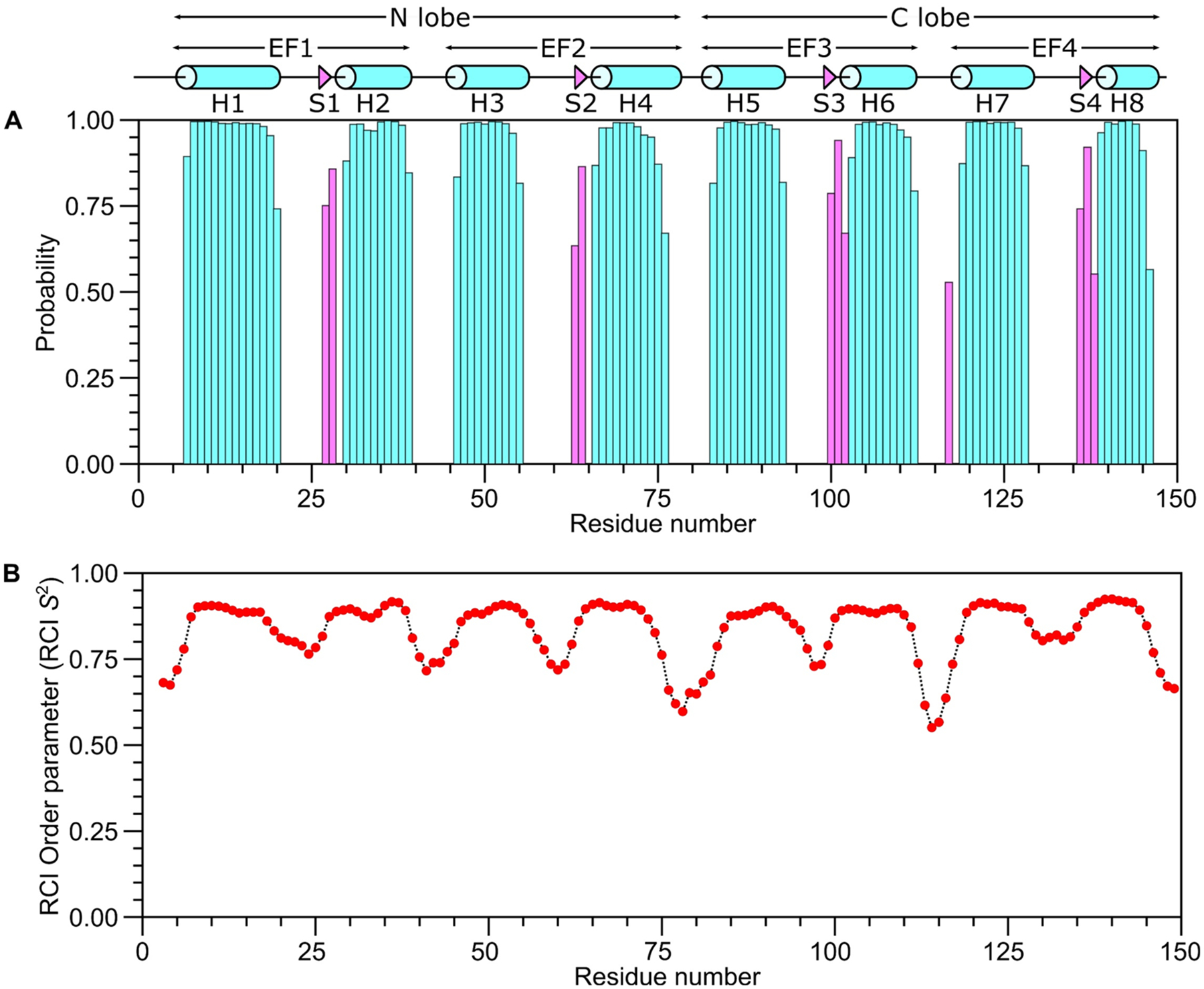
Secondary structure and RCI order parameters of CaM predicted from the assigned backbone chemical shifts. **A** Probability of secondary structural elements (cyan for helix and magenta for strand) and **B** RCI order parameter (RCI-S^2^) of CaM were predicted using TALOS + server (Shen et al. 2009). The wire diagram depicting the secondary structural elements (cylinder for helix and triangle for strand) was obtained from the CaM structure (PDB ID—2VAY (Halling et al. 2009))

The secondary structure of CaM was calculated based on the chemical shift index (Wishart et al. 1992) of each assigned amino acid residue and ANN-Secondary structure prediction using TALOS + (Shen et al. 2009) (Fig. 2). The NMR-derived secondary structure is identical to that found in the CaM crystal structure and contains the following α-helices: H1 (residues 7–20), H2 (residues 30–39), H3 (residues 46–55), H4 (residues 66–76), H5 (residues 83–93), H6 (residues 103–112), H7 (residues 119–128) and H8 (residues 139–146) depicted by cylinders in Fig. 2A. Four short β-strands named S1 (residues 27–28), S2 (residues 63–64), S3 (residues 100–102) and S4 (residues 136–138) are depicted by the triangles in Fig. 2A. NMR-derived distance restraints inferred from NOESY spectra indicate that the observed α-helices and β-strands combine to form 4 EF-hand Ca^2+^ binding motifs (EF1: residues 7–39, EF2: residues 46–76, EF3: residues 83–112 and EF4: residues 119–146) as seen in the crystal structure (Babu et al. 1988). In addition to secondary structure, Talos + calculated the RCI-order parameter (RCI-S^2^) which is a reporter of backbone flexibility (Fig. 2B). The residues with regular secondary structure have the largest RCI-S^2^ values and are rigid, whereas the flexible loops have lower RCI-S^2^ values.

**Fig. 3:**
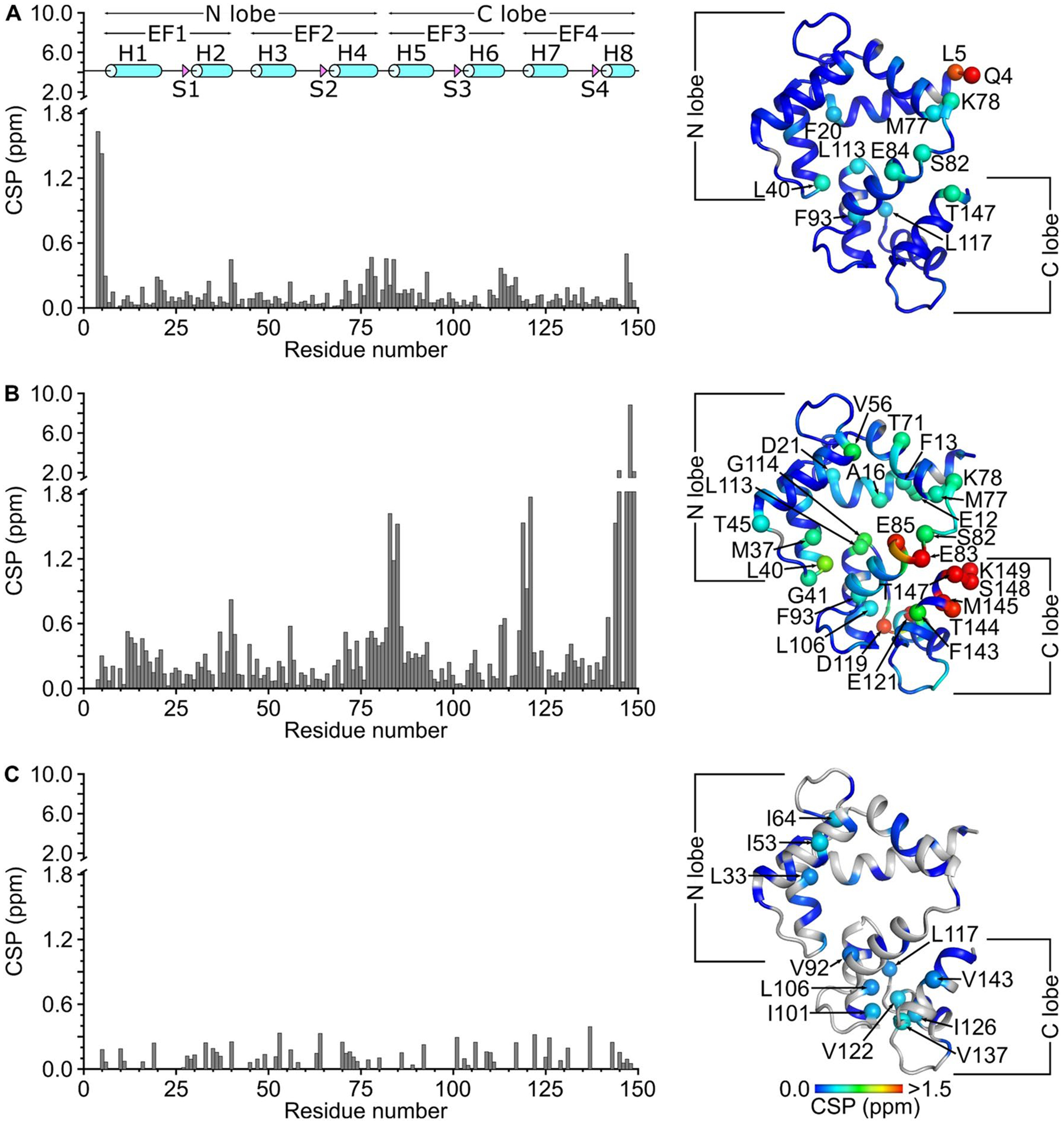
Amide chemical shift perturbation (CSP) for human CaM at neutral pH (BMRB 51289) compared to **A** frog CaM at pH 6.5 and 310 K (BMRB 6541) (Kainosho et al. 2006) and **B** drosophila CaM at pH 6.3 and 320 K (BMRB 547) (Ikura et al. 1990). CSP was calculated as: $CSP=\sqrt{{\left(\Delta H^{N}\right)}^{2}+{\left(\Delta N\right)}^{2}}$. ΔH^N^ and ΔN are the observed difference in the ^1^H^N^ and ^15^N chemical shifts, respectively between CaM at pH 7.0 (BMRB 51289) and CaM at either pH 6.5 (BMRB 6541) or pH 6.3 (BMRB 547). **C** Side-chain methyl chemical shift perturbation was calculated as: $CSP=\sqrt{{\left(\Delta H\right)}^{2}+{\left(\Delta C\right)}^{2}}$. ΔH and ΔC are the observed difference in ^1^H and ^13^C methyl chemical shifts between 51,289 and 6541. CSP values are mapped on to the CaM structure (PDB ID: 2VAY (Halling et al. 2009))

The assigned amide chemical shifts of CaM at neutral pH and 308 K in this study are detectably different from those observed previously at pH 6.5/310 K (Fig. 3A, BMRB 6541) and pH 6.3/320 K (Fig. 3B, BMRB 547). The largest chemical shift variation at pH 6.5 occurs for the amide resonances assigned to exposed residues (Q4, L5, F20, L40, K78, S82, L113, T147 in Fig. 3A). A much larger variation occurs at pH 6.3 and 320 K (E12, L40, E83, E85, D119, E121, T144, M145, K149 in Fig. 3B). The largest variation in Fig. 3B is seen for the C-terminal residues (144, 145, 147, 148 and 149), because residues 144 and 148 are not conserved between human and drosophila CaM. The large variation could also be caused in part by the much higher temperature (320 K) used for BMRB 547 compared to the temperature (308 K) used for the current study (BMRB 51289). Indeed, the CSP of side-chain methyl resonances (which are less sensitive to pH and temperature) are smaller when comparing BMRB 6541 and BMRB 51289 (Fig. 3C). The pH/temperature-dependent variation of amide chemical shifts causes a large baseline error when comparing chemical shifts from BMRB 547 or 6541 vs BMRB 51289. The baseline error (0.25 ppm in Fig. 3A or 0.45 ppm in Fig. 3B) is larger than a typical chemical shift perturbation value obtained when comparing chemical shifts of free CaM versus CaM bound to a target peptide at the same pH (Bej and Ames 2022). Thus, the large pH/temperature-dependent variation in chemical shift causes a significant error when using the CaM chemical shift assignments from BMRB 6541 (Kainosho et al. 2006) or BMRB 547 (Ikura et al. 1990) to calculate a chemical shift perturbation map of a CaM/target complex studied at pH 7.0. Also, some CaM/target complexes are not soluble enough for NMR at acidic pH or higher temperatures, and are more soluble at neutral pH and physiological temperature. Therefore, the NMR resonance assignments of CaM reported here under a set of standard conditions at neutral pH and at 308 K should enable a more accurate analysis of chemical shift perturbation when analyzing future structures of CaM/target complexes at neutral pH.

## Data Availability

The assignments have been deposited to the BMRB under the accession code: 51289.
